# A study on fracture lines of the quadrilateral plate based on fracture mapping

**DOI:** 10.1186/s13018-019-1318-3

**Published:** 2019-09-12

**Authors:** Yun Yang, Chang Zou, Yue Fang

**Affiliations:** Department of Orthopaedics, West China Hospital, Sichuan University, Chengdu, Sichuan People’s Republic of China

**Keywords:** Quadrilateral plate, Acetabulum, Fracture line, Mapping, Judet-Letournel

## Abstract

**Background:**

Quadrilateral plate fractures are a challenging group of acetabular fractures to manage. However, there is little literature that describes the fracture lines of the quadrilateral plate. The aim of this study was to explore the fracture lines of the quadrilateral plate and relevant clinical significance.

**Methods:**

CT data from a series of acetabular fractures were retrospectively analyzed. According to the X-ray, CT, and operative records of the patients, Judet-Letournel classification was carried out for acetabular fractures involving quadrilateral plate. Then, the fracture maps of different types of acetabular fractures in the quadrilateral plate were drawn. To facilitate the characterization of fracture maps, we defined six basic fracture lines.

**Results:**

The fracture lines of the three types of acetabular fractures (double-column fracture, T-type fracture, and anterior column with posterior hemitransverse fracture) mainly included upper transverse lines and upper oblique lines. Although the fracture lines of posterior wall fracture and anterior column fracture were mainly upper transverse lines, the fracture lines of the former were in a low position. The fracture lines of transverse fracture and transverse with posterior wall fracture were similar, both of which were mainly upper oblique lines. The fracture lines of posterior column fractures mainly included posterior vertical lines.

**Conclusions:**

The fracture lines of different types of acetabular fractures have certain regularity respectively. Observation of the fracture lines of the quadrilateral plate based on fracture mapping can help orthopedic surgeons to enhance the understanding of the Judet-Letournel classification, which may have some significant guidance on the choice of operation approach and the design of internal fixation devices.

## Background

Anatomic reduction and stable internal fixation of displaced acetabular fractures has been considered the accepted treatment for these injuries to decrease the rate of post-traumatic arthrosis [1, 2]. Clinical outcomes were correlated with variables including fracture type, the accuracy of reduction, and patient age [3–6].

At present, the most clinically used classification is the Judet-Letournel classification [7, 8], which is based on the column theory and describes fracture anatomy in relation to the anterior and posterior columns. Initial radiographic assessment is with plain radiography using the anteroposterior (AP) pelvis and Judet views. However, some fracture types are easily confused. In addition, less experienced orthopedic surgeons have difficulty in understanding the three-dimensional structure of acetabular fracture and Judet-Letournel classification, which is not conducive to select the appropriate treatment plan.

The accuracy of reduction is closely related to the choice of surgical approach. The most commonly used approaches include Stoppa, ilioinguinal (IL), and Kocher-Langenbeck (KL). However, no single approach can meet the needs of all acetabular fracture types. The choice of surgical approach depends on multiple factors, including the fracture pattern, familiarity with complex anatomical structures, fracture morphology, and surgeon’s individual preference.

Since most types of acetabular fracture (excluding simple anterior wall fracture and posterior wall fracture) involve the quadrilateral plate, we envisioned to simplify the method and observe the fracture lines of the quadrilateral plate to guide preoperative planning. Therefore, we applied fracture map technique to the study of fracture lines of the quadrilateral plate and expected to explore the clinical significance.

## Materials and methods

### Subjects

A retrospective search in the hospital database was conducted for CT data of patients with acetabular fracture between January 2009 and May 2017 at a level I trauma center. Inclusion criteria were (1) age of 18 years or older, (2) fracture involving the quadrilateral plate, (3) complete radiographic assessment, and (4) eight fracture types in Letournel classification. The exclusion criteria were (1) CT images of insufficient quality, (2) severe comminuted fractures that fail to identify the fracture line, and (3) pathological fractures (pelvic bone destruction or bone loss caused by tumor, infection, or metabolic disease).

Before the study began, we had carefully consulted the Ethics Committee and Institutional Review Board of West China Hospital. They suggested that this study did not involve special interventions for patients and we should conduct this study in compliance with the Helsinki Declaration. So, all data was fully anonymized at-source. Given the anonymous nature of the data, the ethics committee waived any requirement for patient informed consent.

### The description of the quadrilateral plate

**Fig. 1:**
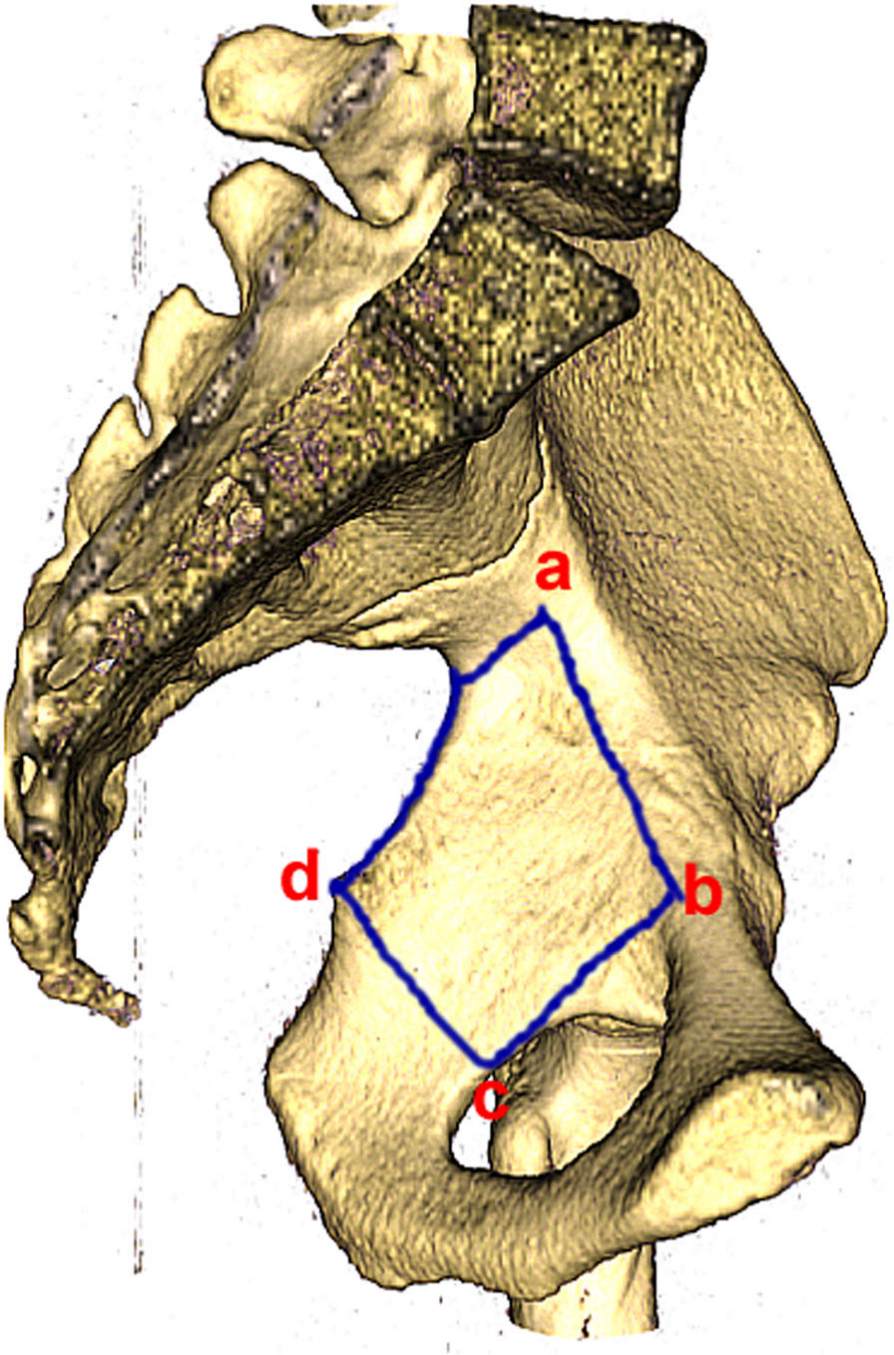
The area enclosed by the four-point connection roughly represented quadrilateral plate

According to our understanding of the quadrilateral plate and from the previous description by ElNahal et al [9], we identified the borders of the quadrilateral plate, which is bound by the greater sciatic foramen posteriorly, the obturator foramen anteriorly, and the pelvic brim superiorly, with a horizontal line joining the ischial spine and the obturator foramen inferiorly. To simplify the process, we took four points of “a, b, c, d” on a pelvic model. These points respectively represented the intersection of the anterior column and the posterior column, obturator groove, rear edge of the obturator ring, and ischial spine. The area enclosed by the four-point connection roughly represented the trapezoidal-shaped area of quadrilateral plate (Fig. 1).

### Fracture mapping

The method of fracture mapping was previously described by Cole and colleagues [10, 11]. In our study, the fracture mapping methodology was modified for quadrilateral plate fractures by reviewing 3DCT images. Subsequently, lines and zones of each respective case were graphically superimposed onto a standard template of an intact left hemipelvis, which resulted in the construction of fracture maps of different types of acetabular fractures in the quadrilateral plate.

**Fig. 2:**
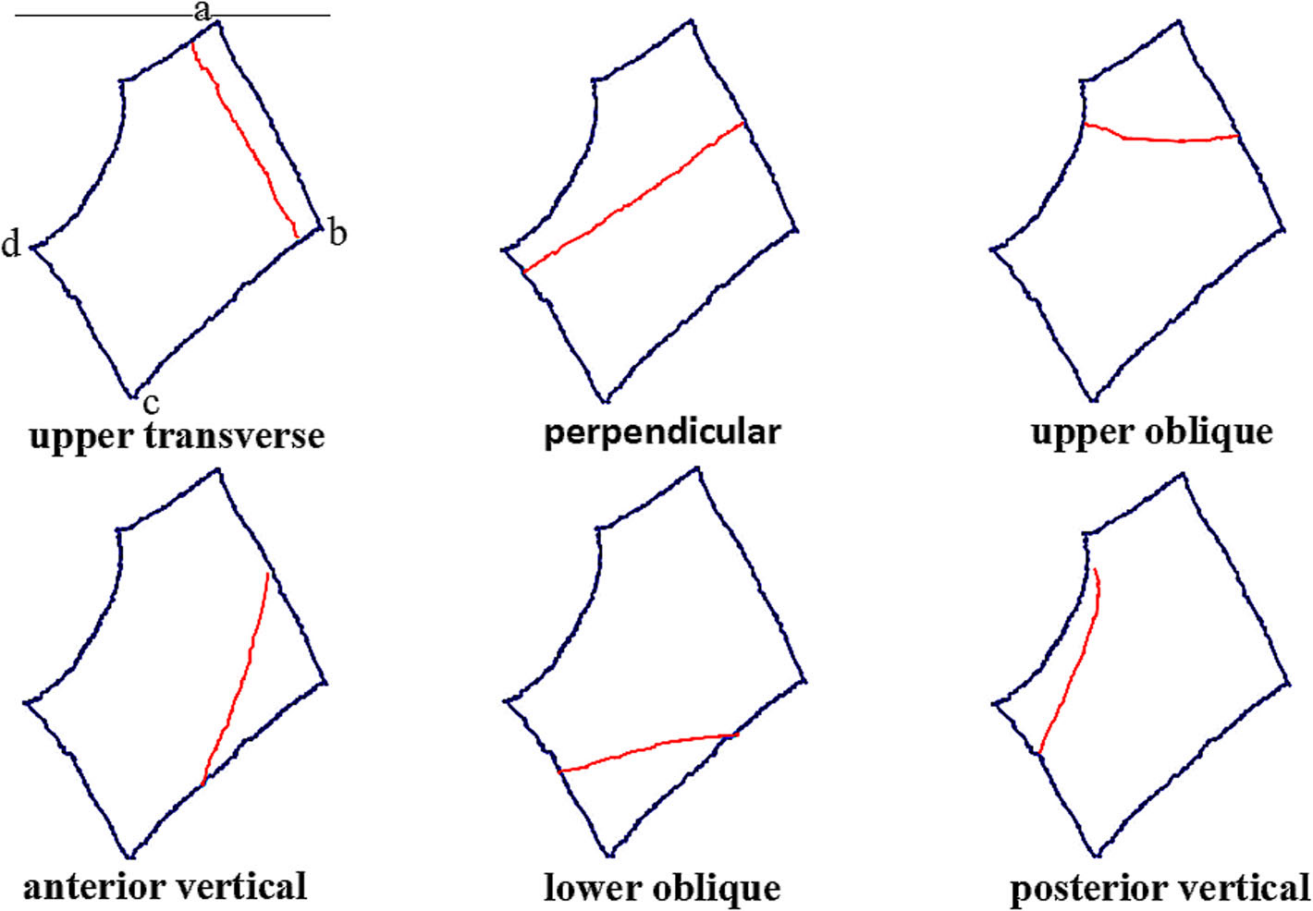
Definition of different fracture lines

For the convenience of understanding, the arcuate line was used as the reference plane to define the fracture lines of the quadrilateral plate. The fracture line through the “ad” side and “bc” side was defined as upper transverse, that through the “ab” side and “cd” side was defined as perpendicular, that through the “ab” side and “ad” side was defined as upper oblique, that through the “bc” side and “cd” side was defined as lower oblique, that through the “ab” side and “bc” side was defined as anterior vertical, and that through the “ad” side and “cd” side was defined as posterior vertical (Fig. 2).

### Analysis of the relationship between fracture lines and the Judet-Letournel classification

Observe the fracture maps of different types of acetabular fractures, then list all possible fracture lines, and assess the relationship between fracture lines and the Judet-Letournel classification qualitatively and quantitatively at last.

### Data analysis

The analysis of the fracture maps was descriptive. Patient characteristics were summarized with frequencies and percentages for categorical variables and with means for continuous variables.

## Results

### Subjects

A total of 208 patients met the inclusion criteria. There were 144 (69%) men and 64 (31%) women included in this study with an average age of 43 years (range, 18–77 years). Two hundred six patients underwent operation, and the remaining two patients were conservatively treated due to poor general condition and severe coronary heart disease. The most common mechanism of injury was a high-energy mechanism—motor vehicle collision. Acetabular fractures involving the quadrilateral plate were classified according to Judet-Letournel classification, which consisted of 59 cases of double-column fracture, 43 cases of transverse with posterior wall fracture, 36 cases of T-type fracture, 26 cases of transverse fracture, 20 cases of anterior column with posterior hemitransverse fracture, 12 cases of posterior column with posterior wall fracture, 9 cases of anterior column fracture, and 6 cases of posterior column fracture (Table 1).

**Table 1:** The demographics of subjects

Variable	
Mean age, year	43
Gender, *n* (%)	
Men	144 (69)
Women	64 (31)
Side of injury, *n* (%)	
Right	96 (46)
Left	109 (52)
Bilateral	3 (2)
Treatment, *n* (%)	
Operative	206 (99)
Conservative	2 (1)
Mechanism of injury, *n* (%)	
Motor vehicle collision	116 (56)
Fall from height	74 (35)
Others	18 (9)
All patients, *n*	208
Letournel classification, *n* (%)	
Double-column	59 (28)
Transverse with posterior wall	43 (20)
T-type	36 (17)
Transverse	26 (12)
Anterior column with posterior hemitransverse	20 (10)
Posterior column with posterior wall	12 (6)
Anterior column	9 (4)
Posterior column	6 (3)
All fractures, *n*	211

### The qualitative description of fracture lines based on fracture maps

**Fig. 3:**
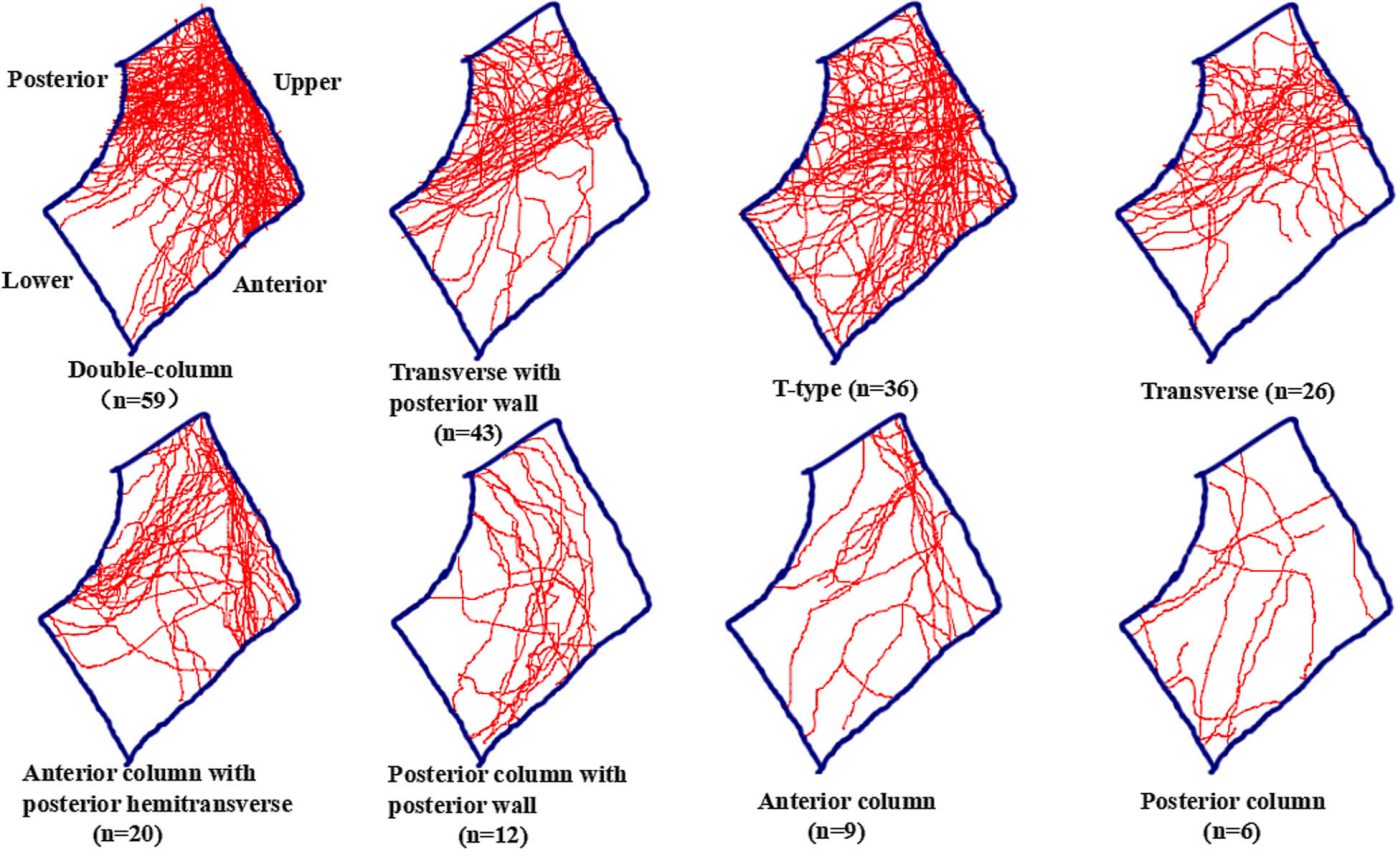
Fracture maps and fracture lines of acetabular fractures involving the quadrilateral plate

Different fracture types have different fracture lines. The details are as follows: the fracture lines of double-column fractures were concentrated on the top and posterior part of the quadrilateral plate, mainly including upper transverse lines and upper oblique lines; the fracture lines of transverse with posterior wall fractures were in the posterior part, mainly including upper oblique lines; the fracture lines of T-type fractures were relatively evenly distributed, mainly including upper oblique lines and upper transverse lines; the fracture lines of transverse fractures were also in the posterior part, mainly including upper oblique lines; the fracture lines of the anterior column with posterior hemitransverse fractures were in the upper and the posterior part, mainly including upper transverse lines and upper oblique lines; the fracture lines of the posterior column with posterior wall fractures were mainly in the middle part, mainly including upper transverse lines; the fracture lines of anterior column fractures were in the upper part, mainly including upper transverse lines; the fracture lines of posterior column fractures were in the infero-posterior part, mainly including posterior vertical lines (Fig. 3).

### The quantitative description of fracture lines in combination with the fracture maps

The fracture lines of different types of acetabular fractures and the number were detailed in Table 2.

**Table 2:** The quantitative relationship between fracture lines and the Judet-Letournel classification

Letournel classification	Number of fracture (*n*)	Fracture lines in the quadrilateral plate (*n*)						Total
		Upper transverse	Perpendicular	Upper oblique	Anterior vertical	Lower oblique	Posterior vertical	
DC	59	50	18	49	14	0	2	133
TPW	43	8	12	29	1	0	1	51
T-type	36	23	7	28	11	1	0	70
T	26	4	6	19	1	0	2	32
AC + PH	20	19	5	14	0	1	0	39
PC + PW	12	9	0	5	1	0	2	17
AC	9	6	4	1	2	1	0	14
PC	6	1	1	2	0	0	4	8
Total	211	120	53	147	30	3	11	364

*DC* double-column, *TPW* transverse with posterior wall, *T* transverse, *AC + PH* anterior column with posterior hemitransverse, *PC + PW* posterior column with posterior wall, *AC* anterior column, *PC* posterior column

## Discussion

The Judet-Letournel classification [7, 8] has been widely accepted for classifying acetabular fractures, communicating with peers, selecting surgical approach, and reporting results [12–14]. This classification is associated with high interobserver and intraobserver reliability when used by orthopedic surgeons with experience in treating acetabular fractures [15]. However, it is more difficult for orthopedic surgeons with less experience to correctly classify acetabular fractures [15, 16].

**Fig. 4:**
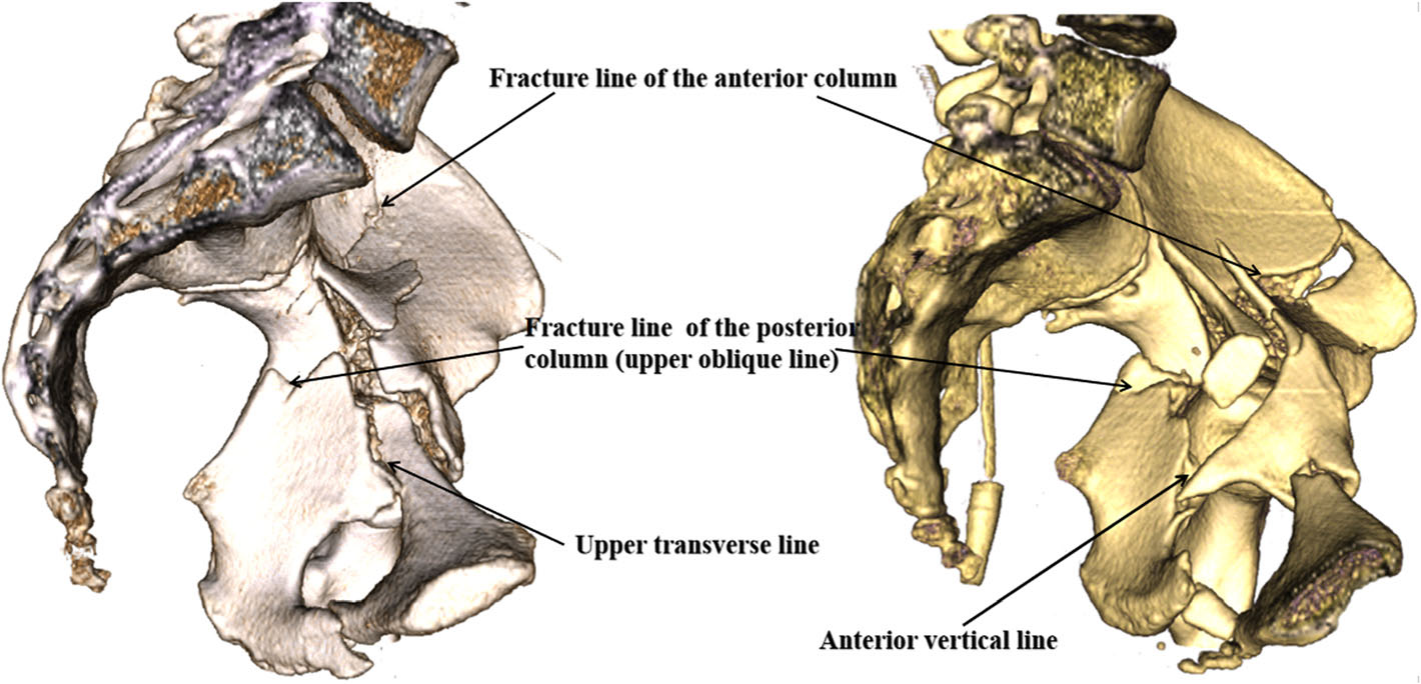
The presentation of double-column fractures in the medial side of the pelvis (including the fractures lines in the quadrilateral plate)

By understanding the relationship between fracture lines and the Judet-Letournel classification, orthopedic surgeons have features of all fracture types in mind without remembering a complex classification system. For example, the fracture lines of double-column fractures mainly include upper transverse lines and upper oblique lines. The fracture line of the anterior column meets the fracture line (upper oblique line) of the posterior column at the border of the true pelvis and then enters the obturator ring by upper transverse or anterior vertical line together, which is consistent with the fracture characteristics of the double-column fractures (Fig. 4).

**Fig. 5:**
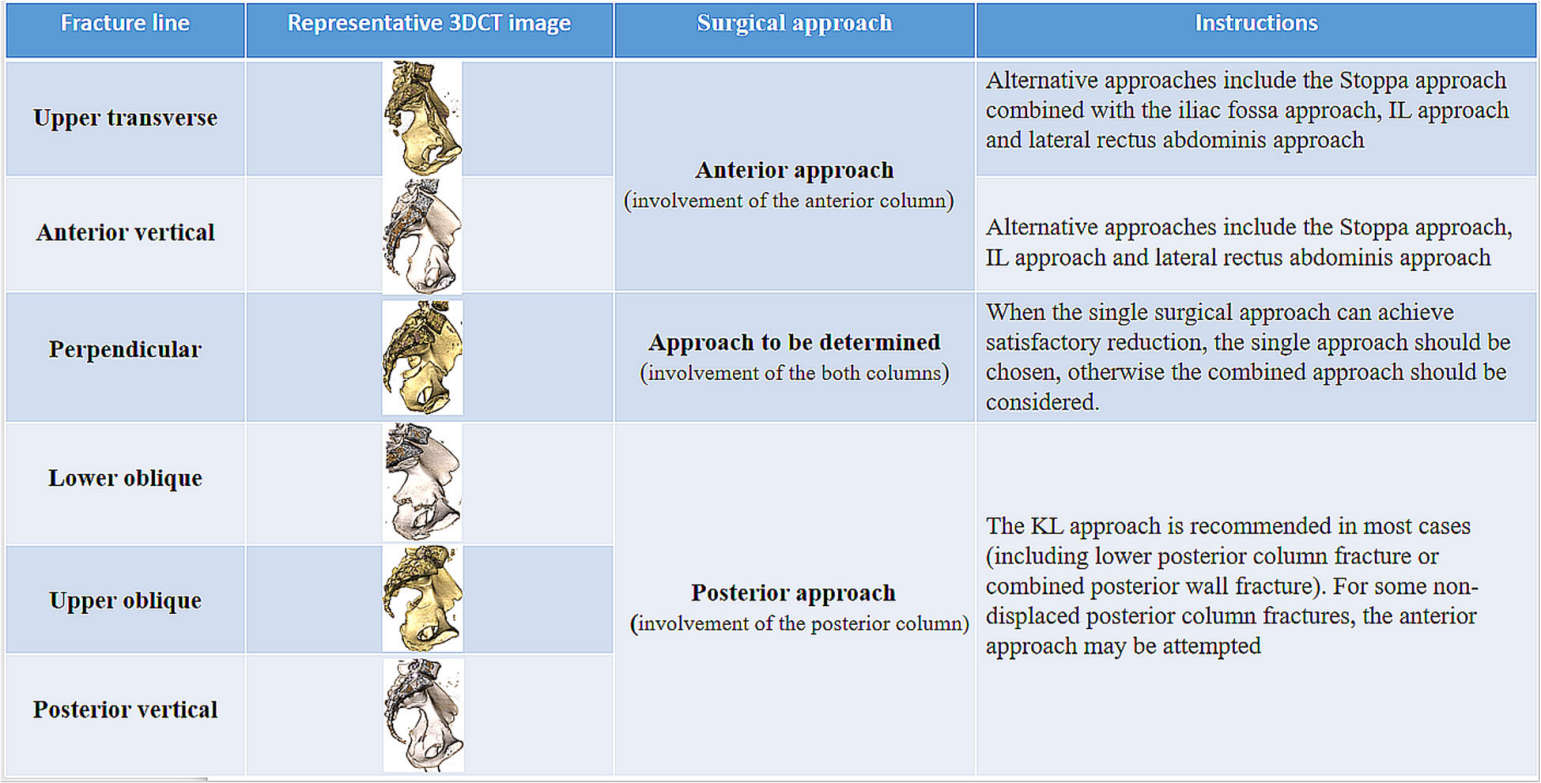
Representative three-dimensional computed tomography image of hemipelvis with acetabular fracture is presented for illustrative purposes. Different surgical approaches are chosen according to different fracture lines

In addition, by observing fracture lines of different types of acetabular fractures and understanding of the pelvic anatomy, it can help orthopedic counterparts to choose the appropriate surgical approach. It should be emphasized that fracture lines of the quadrilateral plate are not the only factor in the selection of surgical approach. In general, anterior approach can be used for fractures involving the anterior column and posterior approach can be used for fractures involving the posterior column. However, if the anterior and posterior columns are involved, we also need to take other factors into account in choosing the surgical approach, such as the direction of the fracture displacement, whether accompanied by posterior wall fracture, surgeon’s individual preference, and fixation way. Based on our understanding and previous summary, we had some experience about the choice of surgical approach. Upper transverse and anterior vertical lines mainly involve the anterior column, which were suitable for the anterior approach. While upper oblique, lower oblique, and posterior vertical line mainly involve the posterior column, the posterior approach was appropriate. In addition, because the perpendicular line involved both columns, the combined approach should be chosen in theory. However, a single approach should be chosen for fractures that can be solved by a single approach (Fig. 5).

Quadrilateral plate fractures are often accompanied by medial migration of the femoral head, especially in osteopenic patients [17, 18]. Fixation of such fractures become technically challenging. The internal fixation devices have screws, plates, and steel wires, among which plates are the most commonly used and the biomechanical stability is relatively good. Although various new internal fixation devices have emerged in endlessly, the long-term efficacy needs to be further evaluated [19–22].

In our study, upper transverse, upper oblique, and perpendicular lines were most common in acetabular fractures involving the quadrilateral plate. Therefore, we have proposed to design an internal fixation system that meets the following requirements: (1) can cover the three most common fracture lines above, (2) can resist the internal movement trend of the quadrilateral plate, (3) can avoid screws entering the hip, and (4) can better serve the bone surface. At present, our study focuses on the measurement of pelvic anatomical parameters and the design of the new plate.

This study had also several limitations. First, the methods and results were descriptive so that others may be suspicious of the interpretation of fracture maps. Second, some hemipelvis images did not match the hemipelvis model perfectly. Third, this requires a CT technician to crop and rotate the 3D reconstruction image of the pelvis to expose the quadrilateral plate. Finally, our analysis did not account for potential variability in function, anatomy, and injury mechanism.

## Conclusions

In conclusion, observation of the fracture line by fracture mapping provides us with a new perspective to study acetabular fractures, which helps orthopedists understand the acetabular fracture classification intuitively and comprehensively. Fracture lines of the quadrilateral plate can provide some guidance for the choice of surgical approach and the design of internal fixation for acetabular fractures.

## Data Availability

This article only includes summarized data from this study. Anonymized datasets (CT) are available from the corresponding author on reasonable request.

## Abbreviations

3DCT: Three-dimensional computed tomography
AP: Anteroposterior
CT: Computed tomography
IL: Ilioinguinal
KL: Kocher-Langenbeck
